# Solubility and Thermodynamic Data of Febuxostat in Various Mono Solvents at Different Temperatures

**DOI:** 10.3390/molecules27134043

**Published:** 2022-06-23

**Authors:** Nazrul Haq, Adel F. Alghaith, Sultan Alshehri, Faiyaz Shakeel

**Affiliations:** Department of Pharmaceutics, College of Pharmacy, King Saud University, Riyadh 11451, Saudi Arabia; nhaq@ksu.edu.sa (N.H.); afalghaith@ksu.edu.sa (A.F.A.); salshehri1@ksu.edu.sa (S.A.)

**Keywords:** dissolution thermodynamics, febuxostat, solubility, hansen solubility parameter

## Abstract

This study examines the solubility and thermodynamics of febuxostat (FBX) in a variety of mono solvents, including “water, methanol (MeOH), ethanol (EtOH), isopropanol (IPA), 1-butanol (1-BuOH), 2-butanol (2-BuOH), ethylene glycol (EG), propylene glycol (PG), polyethylene glycol-400 (PEG-400), ethyl acetate (EA), Transcutol-HP (THP), and dimethyl sulfoxide (DMSO)” at 298.2–318.2 K and 101.1 kPa. The solubility of FBX was determined using a shake flask method and correlated with “van’t Hoff, Buchowski-Ksiazczak *λh*, and Apelblat models”. The overall error values for van’t Hoff, Buchowski-Ksiazczak *λh*, and Apelblat models was recorded to be 1.60, 2.86, and 1.14%, respectively. The maximum mole fraction solubility of FBX was 3.06 × 10^−2^ in PEG-400 at 318.2 K, however the least one was 1.97 × 10^−7^ in water at 298.2 K. The FBX solubility increased with temperature and the order followed in different mono solvents was PEG-400 (3.06 × 10^−2^) > THP (1.70 × 10^−2^) > 2-BuOH (1.38 × 10^−2^) > 1-BuOH (1.37 × 10^−2^) > IPA (1.10 × 10^−2^) > EtOH (8.37 × 10^−3^) > EA (8.31 × 10^−3^) > DMSO (7.35 × 10^−3^) > MeOH (3.26 × 10^−3^) > PG (1.88 × 10^−3^) > EG (1.31 × 10^−3^) > water (1.14 × 10^−6^) at 318.2 K. Compared to the other combinations of FBX and mono solvents, FBX-PEG-400 had the strongest solute-solvent interactions. The apparent thermodynamic analysis revealed that FBX dissolution was “endothermic and entropy-driven” in all mono solvents investigated. Based on these findings, PEG-400 appears to be the optimal co-solvent for FBX solubility.

## 1. Introduction

**Figure 1:**
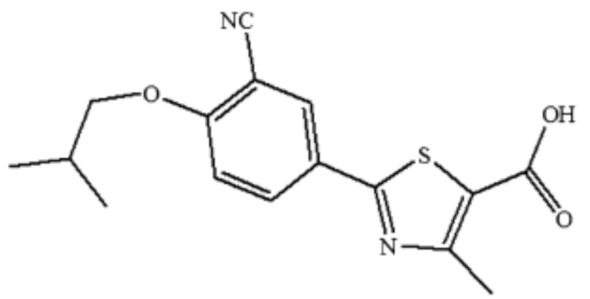
Molecular structure of febuxostat (FBX).

Febuxostat (FBX) (molecular structure: Figure 1; IUPAC name: 2-[3-cyano-4-(2-methylpropoxy)phenyl]-4-methylthiazole-5-carboxylic acid) occurs as a white crystalline powder [1,2]. It is a selective nonpurine inhibitor of xanthine oxidoreductase [3]. It has been recommended for the treatment of hyperuricemia in adults with gout [3,4]. Polymorphism is one of the characteristics of FBX [5]. Three polymorphs (form A, B, and C) and two solvates (BH and D) are among the five distinct forms of FBX [6,7,8]. The most preferred forms of FBX is form A and its crystallization process is difficult to control [7]. A novel crystalline form of FBX (form H) has been identified that has been demonstrated to be stable under a variety of circumstances and is ideal for dosage form design [1].

FBX is a biopharmaceutical classification system (BCS) class II drug with poor solubility in aqueous media and high permeability [2]. Its solubility in water is very poor, which is the main hurdle for its formulation development [9]. The solubility and physicochemical data of FBX are poorly reported in literature [1,9]. The solubility data, solubility parameters, and thermodynamic properties of poorly water-soluble compounds in aqueous and organic solvents are important for various industrial applications [10,11,12,13]. The solubility of FBX in water at 310.2 K has been reported [9]. The solubility of FBX in four different organic solvents such as methanol (MeOH), ethanol (EtOH), acetone, and ethyl acetate (EA) at 293.15–328.15 K and 101.1 kPa has been reported well in the literature [1].

Other researched mono solvents such as isopropanol (IPA), 1-butanol (1-BuOH), 2-butanol (2-BuOH), ethylene glycol (EG), propylene glycol (PG), polyethylene glycol-400 (PEG-400), Transcutol-HP (THP), and dimethyl sulfoxide (DMSO) have not had their solubility and other physicochemical data published. As a result, new solubility and physicochemical data for FBX in various mono solvents, including water, MeOH, EtOH, IPA, 1-BuOH, 2-BuOH, EG, PG, PEG-400, THP, EA, and DMSO at 298.2–318.2 K and 101.1 kPa, are reported in this study. “Apparent thermodynamic analysis” was carried out to evaluate the dissolution behavior of FBX in various mono solvents. The solubility and physicochemical data of FBX obtained in this research could be used in “purification, recrystallization, drug discovery, pre-formulation studies, and formulation development” of FBX.

## 2. Materials and Methods

### 2.1. Materials and Reagents

The FBX (form H) standard drug was procured from “E-Merck (Darmstadt, Germany)”. EG, PG, PEG-400, EA, and DMSO were procured from “Sigma Aldrich (St. Louis, MO, USA)”. MeOH, EtOH, IPA, 1-BuOH, and 2-BuOH were obtained from “E-Merck (Darmstadt, Germany)”. The Milli-Q unit provided purified water. The details about each material are summarized in Supplementary Table S1.

### 2.2. Determination of FBX Using High-Performance Liquid Chromatography (HPLC) Method

A validated HPLC method was used to analyze FBX in solubility samples. The quantitation of FBX was performed via ultra-violet (UV) detector at a wavelength of 354 nm. The entire estimation of FBX was performed at 298.2 K utilizing “HPLC system (Waters, Milford, MA, USA)”. The column “Nucleodur (150 × 4.6 mm, 5 μm) reverse-phase C_18_ column” was used for the chromatographic analysis of FBX. The binary mixture of EtOH and EA (50:50, % *v*/*v*) was used as the greener solvent system under the flow rate of 1.0 mL min^−1^. Prior to use, the greener solvent combination was freshly produced, filtered through nylon filter paper with a pore size of 0.45 µm, and degassed. The injection volume was set at 20 µL. The FBX calibration curve was created by plotting the FBX concentrations against the measured HPLC response. In the range of 1–100 µg g^−1^, the calibration plot of FBX was linear, with a determination coefficient (R^2^) of 0.9979. The regression line equation for FBX was *y* = 46,266*x* + 14,848; where *x* and y represent the FBX concentration and observed HPLC response, respectively.

### 2.3. Solid Phase Characterization of FBX

For pure FBX and equilibrated FBX (the solid recovered from bottom phase of equilibrated sample), Fourier transforms infrared (FTIR) spectroscopy and powder X-ray diffraction (PXRD) analyses were used to characterize the solid phases. Slow evaporation was used to recover the equilibrated FBX from water [12,13]. For FTIR analysis, the absorption spectra were obtained in the range of 300–4000 cm^−1^ using the potassium bromide disc technique as reported in literature [14]. For PXRD measurement, the samples were analyzed by “Ultima IV Diffractometer (Rigaku Inc., Tokyo, Japan)” equipped with “Cu–Kα radiation 1.5406 Å”. With a step size of 0.02°, both pure and equilibrated FBX samples were examined in the 2θ range of 2–60° [13]. The FTIR and PXRD analyses were used to investigate the probable transformations of FBX into other physical states, such as polymorphs, solvates, and hydrates, among others.

### 2.4. FBX Solubility Measurement

At 298.2–318.2 K and 101.1 kPa, the solubility of FBX in several mono solvents was measured using an experimental approach proposed by “Higuchi and Connors” [15]. The extra FBX was mixed with the known amount of each mono solvent. The obtained suspensions were vortexed for 10 min. All of the samples were shaken at 100 rpm for 72 h in a “WiseBath^®^ WSB Shaking Water Bath (Model WSB-18/30/-45, Daihan Scientific Co., Ltd., Seoul, Korea)” [16,17]. Each sample was obtained, filtered, and centrifuged at 5000 rpm for 30 min after equilibrium was reached (equilibrium time = 72 h). The supernatant was obtained, diluted (as needed), and utilized to determine the amount of FBX in the sample using the HPLC-UV technique at a wavelength of 354 nm. The “experimental mole fraction solubility (*x*_e_)” of FBX was computed by the following equation [18,19]:

(1) $$x_{e}=\frac{m_{1}/M_{1}}{m_{1}/M_{1}+m_{2}/M_{2}}$$

where, *m*_1_ = FBX mass, *m*_2_ = solvent mass, *M*_1_ = FBX molar mass, and *M*_2_ = solvent molar mass.

### 2.5. Computation of Solubility Parameters

Drug compounds with similar solubility parameters could reach the maximum solubility in the sample matrices under standard conditions [20]. As a result, this study estimated different solubility parameters for FBX and several mono solvents. The following equation was used to calculate the total “Hansen solubility parameter (HSP)” of FBX [20,21,22]:

(2) $$\delta^{2}=\delta_{d}^{2}+\delta_{p}^{2}+\delta_{h}^{2}$$

where, “*δ* = total HSP of FBX; *δ*_d_ = dispersion HSP of FBX; *δ*_p_ = polar HSP of FBX, and *δ*_h_ = hydrogen-bonded HSP of FBX”.

The “HSPiP software (version 4.1.07, Louisville, KY, USA)” was used to calculate the values of *δ, δ*_d_, *δ*_p_, and *δ*_h_ for FBX. These values were determined by putting the simplified molecular input line entry system (SMILES) of FBX into HSPiP software. The SMILES of FBX was taken from its PubChem data. However, the values of *δ, δ*_d_, *δ*_p_, and *δ*_h_ for various mono solvents were taken from reference [12].

The following equation was used to calculate the “van Krevelen and Hoftyzer solubility parameter (∆$\bar{\delta }$)” [23]:

(3) $$∆\bar{\delta }=\lceil \left(\delta_{d}{}_{2}^{2}-\delta_{d}{}_{1}^{2}\right)+\left(\delta_{p}{}_{2}^{2}-\delta_{p}{}_{1}^{2}\right)+{\left(\delta_{h}{}_{2}^{2}-\delta_{h}{}_{1}^{2}\right)\rceil }^{1/2}$$

Subscripts 1 and 2 denote the specific mono solvent and FBX, respectively. According to the literature if ∆$\bar{\delta }$ < 5.0 MPa^1/2^, the solubility of the solute in the mono solvent will be higher [23,24].

The following equation was used to compute the “three dimensional (3D) solubility parameter space (*R*_a_)” [25,26]:

(4) $$Ra^{2}=4{\left(\delta_{d2}-\delta_{d1}\right)}^{2}+{\left(\delta_{p2}-\delta_{p1}\right)}^{2}+{\left(\delta_{h2}-\delta_{h1}\right)}^{2}$$

According to literature, the solubility of FBX in mono solvent will be higher if *R*_a_ < 5.6 MPa^1/2^ [25].

The “Greenhalgh’s solubility parameter (∆*δ*)” was computed using the following equation [27]:

(5) $$∆\delta =\delta_{2}-\delta_{1}$$

According to the literature, the solubility of FBX in mono solvent will be higher if ∆*δ* < 7.0 MPa^1/2^ [21,27].

### 2.6. Ideal Solubility of FBX and Solute-Solvent Interactions

The following equation was used to compute an “ideal solubility (*x*^idl^)” of FBX at 298.2–318.2 K [28]:

(6) $$\ln\;x^{\mathrm{idl}}=\frac{-∆H_{\mathrm{fus}}\left(T_{\mathrm{fus}}-T\right)}{RT_{\mathrm{fus}}T}+\left(\frac{∆C_{p}}{R}\right)[\frac{T_{\mathrm{fus}}-T}{T}+\ln\left(\frac{T}{T_{\mathrm{fus}}}\right)]\;$$

*R* is the universal gas constant, *T*_fus_ is the FBX fusion temperature, ∆*H*_fus_ is the FBX fusion enthalpy, and ∆*C*_p_ is the difference between FBX’s solid phase and liquid state molar heat capacity. The following equation was used to compute the ∆*C*_p_ for FBX [28,29]:

(7) $$∆C_{p}=\frac{∆H_{\mathrm{fus}}}{T_{\mathrm{fus}}}$$

From reference [2], the *T*_fus_ and ∆*H*_fus_ values for FBX were derived as 486.53 K and 27.58 kJ mol^−^^1^, respectively. Using Equation (7), the ∆*C*_p_ for FBX was estimated to be 56.68 J mol^−1^ K^−1^. Finally, using Equation (6), the *x*^idl^ values for FBX were calculated.

The following equation was used to calculate the “activity coefficients (*γ*_i_)” for FBX in several mono solvents at 298.2–318.2 K [28,30]:

(8) $$\gamma_{i}=\frac{x^{\mathrm{idl}}}{x_{e}}$$

Based on the computed *γ*_i_ values of FBX at 298.2–318.3 K, FBX-solvent molecular interactions were estimated.

### 2.7. FBX Solubility Correlation Using Computational Approaches

For practical validations, computational analysis of experimental solubility of solutes is critical [31,32]. As a consequence, the experimental solubility of FBX was correlated with “van’t Hoff, Buchowski-Ksiazczak *λh*, and Apelblat models” [21,33,34,35,36,37]. The following equation was used to calculate the “Apelblat model solubility (*x*^Apl^)” of FBX [33,34]:

(9) $$\ln\;x^{\mathrm{Apl}}=A+\frac{B}{T}+C\ln\left(T\right)$$

*A*, *B*, and *C* are the model parameters obtained from the experimental FBX solubility data reported in Table 1 using “nonlinear multivariate regression analysis” [21]. The correlation between *x*_e_ and *x*^Apl^ values of FBX was carried out in terms of “root mean square deviation (*RMSD*) and *R*^2^”. The *RMSD* of FBX was computed using its reported equation [11].

The following equation was used to calculate the “van’t Hoff model solubility (*x*^van’t^)” of FBX [21]:

(10) $$\ln\;x^{\mathrm{van}’t}=a+\frac{b}{T}$$

Here, *a* and *b* are the “van’t Hoff model” parameters, which were derived using “least square approach” [37].

The following equation was used to calculate the “Buchowski-Ksiazczak *λh* solubility (*x^λh^*)” for FBX [35,36]:

(11) $$\ln\;[1+\frac{\lambda \left(1-x^{\lambda h}\right)}{x^{\lambda h}}]=\lambda h\;[\frac{1}{T}-\frac{1}{T_{\mathrm{fus}}}]$$

Here, *λ* and *h* are the adjustable Buchowski-Ksiazczak *λh* model parameters.

### 2.8. Thermodynamic Evaluation

“Apparent thermodynamic analysis” was utilized to determine the thermodynamic characteristics of FBX in several mono solvents. Three different parameters, including “apparent standard enthalpy (Δ_sol_*H*^0^), apparent standard Gibbs energy (Δ_sol_*G*^0^), and apparent standard entropy (Δ_sol_*S*^0^)” for FBX were computed using “van’t Hoff and Krug et al. analysis” [30,38,39]. The “Δ_sol_*H*^0^” for FBX in various mono solvents was computed at the “mean harmonic temperature (*T*_hm_)” of 308 K utilizing “van’t Hoff analysis” using the following equation [30,39]:

(12) ∂ln xe∂1T−1ThmP=−∆solH0R

**Figure 2:**
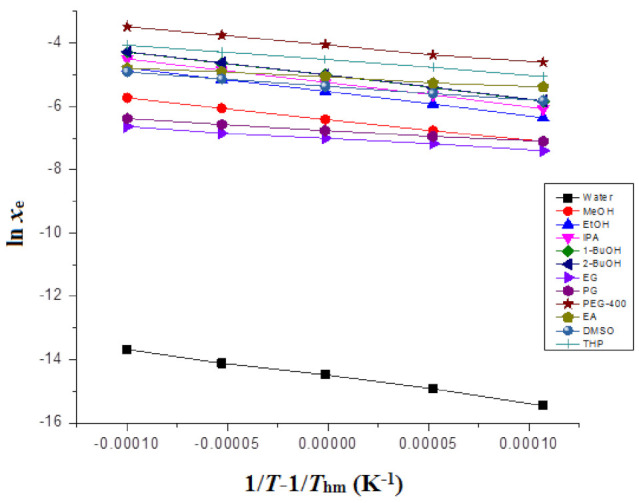
van’t Hoff plots for FBX plotted between ln *x*_e_ and 1/*T* − 1/*T*_hm_ for FBX in different mono solvents.

The “Δ_sol_*H*^0^” for FBX was derived from “van’t Hoff” plots graphed between ln *x*_e_ values of FBX and 1T−1Thm. The van’t Hoff plots for FBX in various mono solvents are included in Figure 2.

The “Δ_sol_*G*^0^” for FBX dissolution in various mono solvents was also computed at *T*_hm_ = 308 K utilizing “Krug et al. analysis” using the following Equation (13) [38]:

(13) $${∆}_{\mathrm{sol}}G^{0}=-RT_{\mathrm{hm}}\times \mathrm{intercept}\;\;\;\;\;\;\;\;\;\;\;\;\;\;\;\;\;\;$$

Here, the intercept values for FBX in various mono solvents were derived from “van’t Hoff plots” included in Figure 2.

The following equation was used to calculate the “Δ_sol_*S*^0^” for FBX dissolution [30,38,39]:

(14) $$\;\;\;\;\;\;\;\;\;\;\;\;\;\;\;\;\;\;\;\;{∆}_{\mathrm{sol}}S^{0}=\frac{{∆}_{\mathrm{sol}}H^{0}-{∆}_{\mathrm{sol}}G^{0}}{T_{\mathrm{hm}}}\;\;\;\;\;\;\;\;\;\;\;\;\;\;\;\;\;\;\;\;\;\;\;\;\;\;\;\;\;\;\;\;\;$$

### 2.9. Statistical Evaluation

“Kruskal-Wallis analysis” was used as a statistical test, followed by “Denn’s test”. In this test, “GraphpadInstat software (San Diego, CA, USA)” was employed. A significant value was defined as a one with a *p* value of less than 0.05.

## 3. Results and Discussion

### 3.1. Solid Phase Characterization of FBX

**Figure 3:**
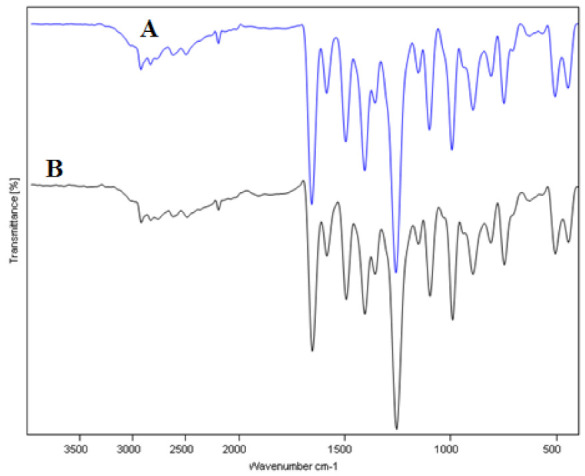
Fourier transforms infra-red (FTIR) spectra of (**A**) pure FBX and (**B**) equilibrated FBX recovered from water.

**Figure 4:**
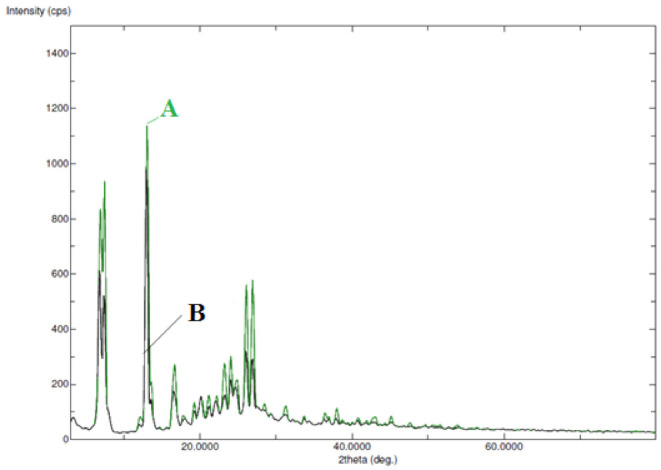
Powder X-ray diffraction (PXRD) spectra of (**A**) pure FBX and (**B**) equilibrated FBX recovered from water.

Form A, form B, form C, and form H, for example, are polymorphic states of the FBX [5,6]. As a result, FTIR and PXRD spectral analysis were used to characterize the solid phase of FBX (form H) in pure and equilibrated samples recovered from water. Figure 3 depicts the FTIR spectra of pure and equilibrated FBX. Pure FBX (form H) FTIR spectra revealed several FBX characteristic peaks at various wave numbers, showing the crystalline nature of pure FBX (Figure 3A). The FTIR spectra of equilibrated FBX recovered from water also revealed identical FBX features peaks at varied wave numbers (Figure 3B), showing that equilibrated FBX is crystalline. Figure 4 depicts the PXRD spectra of pure and equilibrated FBX. The PXRD spectra of pure FBX (form H) revealed multiple crystalline peaks of FBX at different 2θ values, showing that pure FBX is crystalline (Figure 4A). The PXRD spectra of equilibrated FBX recovered from water showed identical features peaks of FBX at various 2θ values (Figure 4B), showing that equilibrated FBX is crystalline. Overall, the FTIR and PXRD spectra revealed that following equilibrium, FBX (form H) was not converted into solvates, polymorphs, or hydrates. It was also expected that FBX will also be retained crystalline in other mono solvents as the experimental conditions were similar for other mono solvents.

### 3.2. Measured Solubility Data of FBX

**Figure 5:**
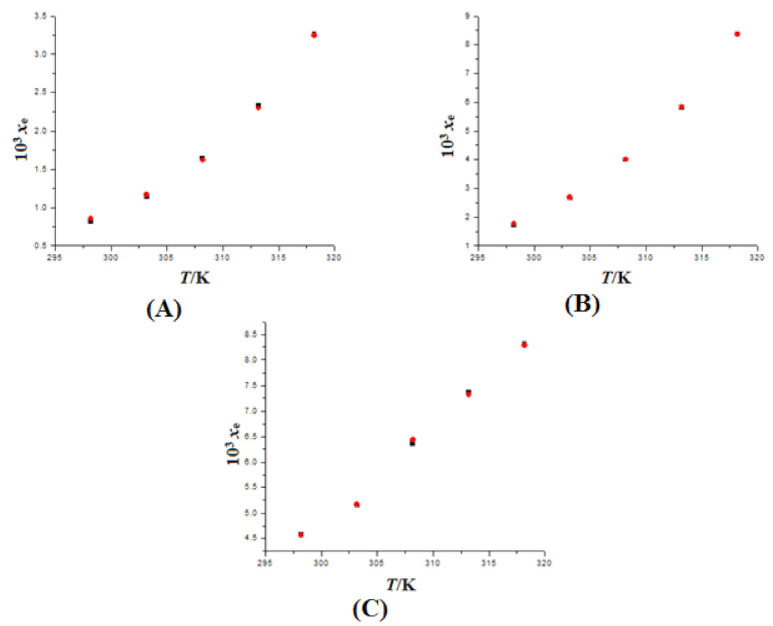
Comparison of mole fraction solubility values of FBX in (**A**) MeOH, (**B**) EtOH, and (**C**) EA with literature values at 298.2–318.2 K; the symbol 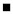 indicates the experimental mole fraction solubilities of FBX in (**A**) MeOH, (**B**) EtOH, and (**C**) EA and the symbol 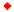 indicates the literature solubilities of FBX in (**A**) MeOH, (**B**) EtOH, and (**C**) EA taken from reference [1].

The measured solubility values of FBX in various mono solvents at 298.2–318.2 K and 101.1 kPa are summarized in Table 1. The solubility of FBX in IPA, 1-BuOH, 2-BuOH, EG, PG, PEG-400, THP, and DMSO, has yet to be determined. At 310.2 K, the saturated solubility of FBX in water was 10.8 mg L^−1^ (converted to 6.15 × 10^−7^ in mole fraction) [9]. At 310.2 K, the mole fraction solubility of FBX was not measured directly in the present study. At 310.2 K, however, the mole fraction solubility of FBX was derived from the interpolation of graph constructed between ln *x*_e_ and 1/*T* and derived to be 6.23 × 10^−7^. The recorded value was closed to the reported value of FBX in water. At 298.2–318.2 K, the mole fraction solubility values of FBX in MeOH, EtOH, and EA have also been published [1]. Figure 5A–C provide a graphical comparison of observed and literature solubility values of FBX in MeOH, EtOH, and EA at 298.2–318.2 K, respectively. The data presented in Figure 5 showed good correlation of measured solubility data of FBX in MeOH, EtOH, and EA with those reported in literature [1]. These findings revealed that the experimental solubility data for FBX were in good accord with the published values [1,9].

Table 1 summarizes the findings, which show that the solubility of FBX increased considerably with increasing temperature in all mono solvents examined (*p* < 0.05) and was in good agreement with prior research [16,17,18]. The order of FBX solubility in different mono solvents was PEG-400 (3.06 × 10^−2^) > THP (1.70 × 10^−2^) > 2-BuOH (1.38 × 10^−2^) > 1-BuOH (1.37 × 10^−2^) > IPA (1.10 × 10^−2^) > EtOH (8.37 × 10^−3^) > EA (8.31 × 10^−3^) > DMSO (7.35 × 10^−3^) > MeOH (3.26 × 10^−3^) > PG (1.88 × 10^−3^) > EG (1.31 × 10^−3^) > water (1.14 × 10^−6^) at 318.2 K. Since PEG-400 has the maximum solubility of FBX compared to the other mono solvents tested, it may be the optimal solvent for FBX solubility.

### 3.3. Determination of HSPs

Different HSPs for FBX were determined using “HSPiP software”. The HSPs of different mono solvents were derived from reference [12]. The values of HSPs are summarized in Table 2. FBX was found to have a *δ* value of 21.70 MPa^1/2^, indicating that it had a low polarity. Seven mono solvents such as IPA (*δ* = 22.30 MPa^1/2^), 1-BuOH (*δ* = 22.90 MPa^1/2^), 2-BuOH (*δ* = 20.80 MPa^1/2^), EA (*δ* = 18.10 MPa^1/2^), 1-DMSO (*δ* = 23.60 MPa^1/2^), THP (*δ* = 21.40 MPa^1/2^), and PEG-400 (*δ* = 18.90 MPa^1/2^) were discovered to exhibit similar *δ* values (lower polarities) and acceptable for FBX solubility. Water had a *δ* value of 47.80 MPa^1/2^, indicating that it was not suited for FBX solubility due to its greater polarity. It was discovered that if ∆$\bar{\delta }$ is <5.0 MPa^1/2^, the biomolecule’s solubility in the particular solvent will be greater [23,24]. The ∆$\bar{\delta }$ value was found to be ≥5.0 MPa^1/2^ in all mono solvents investigated, showing that FBX is insoluble in all mono solvents according to this idea. It has also been discovered that if the value of *R*_a_ is <5.6 MPa^1/2^ the biomolecule’s solubility in the particular solvent will be greater [25,26]. The *R*_a_ values in three mono solvents, EA (*R*_a_ = 7.37 MPa^1/2^), THP (*R*_a_ = 7.81 MPa^1/2^), and DMSO (*R*_a_ = 7.97 MPa^1/2^), were found to be closed with 5.6 MPa^1/2^, showing that FBX is soluble in EA, THP, and DMSO according to this idea. However, the *R*_a_ values in other mono solvents were found to be much higher than 5.6 MPa^1/2^, indicating the insolubility of FBX in other mono solvents according to this concept. According to the Greenhalgh’s theory, the solubility of the biomolecule in the particular solvent will be higher if ∆*δ* is <7.0 MPa^1/2^. However, the value of ∆*δ* > 10.0 MPa^1/2^ has been proposed for the insolubility of biomolecule [27]. The ∆*δ* value was determined to be maximum in water (∆*δ* = 26.10 MPa^1/2^), indicating the complete insolubility of FBX in water. While, the ∆*δ* value was determined to be lower in THP (∆*δ* = 0.30 MPa^1/2^), IPA (∆*δ* = 0.60 MPa^1/2^), 2-BuOH (∆*δ* = 1.10 MPa^1/2^), 1-BuOH (∆*δ* = 1.20 MPa^1/2^), DMSO (∆*δ* = 1.90 MPa^1/2^), PEG-400 (∆*δ* = 2.80 MPa^1/2^), EA (∆*δ* = 3.60 MPa^1/2^), and EtOH (∆*δ* = 3.70 MPa^1/2^), indicating the complete solubility of FBX in all of these mono solvents according to this concept [27].

### 3.4. Determination of Solute-Solvent Interactions

Table 1 summarizes the *x*^idl^ values for FBX. At 298.2–318.2 K, the *x*^idl^ values for FBX were found to be 3.55 × 10^−2^ to 5.52 × 10^−2^. For FBX, the *x*^idl^ values were found to be substantially greater than the *x*_e_ values in water (*p* < 0.05). The *x*^idl^ values of FBX, on the other hand, were found to be near to its *x*_e_ values in PEG-400. PEG-400 was shown to be appropriate for the solubility of FBX based on these findings. The *γ*_i_ values for FBX in different mono solvents at 298.2–318.2 K are summarized in Table 3. Compared to other mono solvents examined, the *γ*_i_ values for FBX were found to be substantially greater in water. With increasing temperature, the *γ*_i_ values of FBX in the mono solvents examined decreased significantly (*p* < 0.05). The *γ*_i_ values for FBX were found to be low in PEG-400, THP, and EA. Based on these results, the maximum solute-solvent interactions were observed in FBX-PEG-400, FBX-THP, and FBX-EA compared to other FBX-solvent combination.

### 3.5. FBX Solubility Correlation Using Computational Approaches

**Figure 6:**
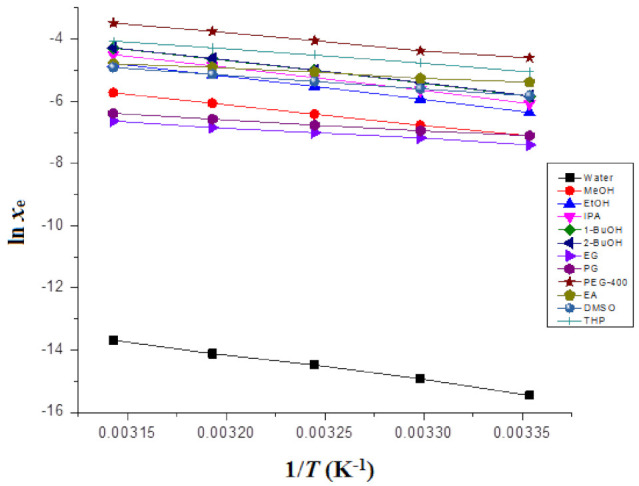
Correlation of experimental FBX solubilities with the “Apelblat model” in several mono solvents as a function of 1/*T*; symbols denote the experimental FBX solubility data, whereas solid lines denote the “Apelblat model” FBX solubility data.

Three computational models namely, “van’t Hoff, Apelblat, and Buchowski-Ksiazaczak *λh* models” were employed to link FBX experimental solubility data in this study [21,33,34,35,36,37]. Figure 6 shows the data for the graphical correlation between *x*_e_ and *x*^Apl^ values of FBX in several mono solvents against 1/*T*, which showed a high correlation between the *x*_e_ and *x*^Apl^ data of FBX in several mono solvents. The results of Apelblat model computation are included in Table 4. The overall *RMSD* for FBX was determined to be 1.14%. *R*^2^ values for FBX in various mono solvents range from 0.9919 to 0.9999. The low *RMSD* values and higher *R*^2^ values revealed that the experimental solubility data of FBX in the mono solvents was well correlated with the Apelblat model.

Figure S1 shows the data for the graphical correlation between *x*_e_ and *x*^van’t^ values of FBX in several mono solvents against 1/*T*, which also showed a high correlation between *x*_e_ and *x*^van’t^ values of FBX in all mono solvents. The results for the van’t Hoff model computation for FBX in various mono solvents are summarized in Table 5. The overall *RMSD* for FBX in various mono solvents was determined to be 1.60%. *R*^2^ values for FBX in several mono solvents range from 0.9920 to 0.9998. The low *RMSD* values and higher *R*^2^ values again revealed that the experimental solubility data of FBX in mono solvents was well correlated with “van’t Hoff model”.

Table 6 summarizes the findings of the Buchowski-Ksiazaczak *λh* computation for FBX in numerous mono solvents. In numerous mono solvents, the overall *RMSD* for FBX was found to be 2.86%. The low *RMSD* values again showed a high correlation of experimental solubility data of FBX in numerous mono solvents with “Buchowski-Ksiazaczak *λh* model”. Overall, all three computational models performed well in the solubility correlation of FBX.

### 3.6. Apparent Thermodynamic Studies

The Δ_sol_*H*^0^ values for FBX in various mono solvents were determined from the van’t Hoff graphs, included in Figure 2. The results of apparent thermodynamic studies of FBX in various mono solvents are listed in Table 7. The FBX Δ_sol_*H* values in numerous mono solvents recorded as positive values in the range of 24.50–68.79 kJ mol^−1^. The FBX Δ_sol_*G*^0^ values in numerous mono solvents were also recorded positive values in the range of 10.38–37.21 kJ mol^−1^. The FBX Δ_sol_*G*^0^ values were found to be lowest in PEG-400 and highest in water, which could be attributed to FBX solubility being highest in PEG-400 and lowest in water, respectively. The positive values of Δ_sol_*H*^0^ for FBX suggested that FBX dissolution was an endothermic in the mono solvents examined [40,41]. The FBX Δ_sol_*S*^0^ values in numerous mono solvents were also recorded as positive values in the range of 37.30–157.6 J mol^−1^ K^−1^, suggesting an entropy-driven FBX dissolution in all mono solvents examined [40]. Based on the positive values of Δ_sol_*H* and Δ_sol_*S*^0^, the FBX dissolution was considered to be an endothermic and entropy-driven in all mono solvents examined [40,41].

## 4. Conclusions

The solubility values, HSPs, and thermodynamics properties of FBX in various mono solvents were investigated. FTIR and PXRD spectral analyses validated the solid state form of FBX, which revealed no alteration of FBX after equilibrium. The results on FBX solubility was strongly associated with the “van’t Hoff, Apelblat, and Buchowski-Ksiazczak *λh* models”. The solubility of FBX increased with increasing temperature in all mono solvents examined. The order of solubility of FBX in various mono solvents was PEG-400 > THP > 2-BuOH > 1-BuOH > IPA > EtOH > EA > DMSO > MeOH > PG > EG > water at 318.2 K. When comparing FBX-PEG-400, FBX-THP, and FBX-EA to other combinations of FBX and mono solvent, the findings of activity coefficients showed that FBX-PEG-400, FBX-THP, and FBX-EA had the most molecular interactions. Thermodynamic study indicated an “endothermic and entropy-driven dissolution” of FBX in all mono solvents examined. Based on overall results, PEG-400 has been selected as the best cosolvent for the solubility of FBX. As a consequence, PEG-400 can be used as a potential co-solvent in pre-formulation studies and formulation development of FBX.

## Figures and Tables

**Table 1:** Experimental solubilities (*x*_e_) and ideal solubilities (*x*^idl^) of febuxostat (FBX) in mole fraction in various mono solvents (MS) at 298.2–318.2 K and 101.1 kPa ^a^.

MS	*x*_e_				
	*T* = 298.2 K	*T* = 303.2 K	*T* = 308.2 K	*T* = 313.2 K	*T* = 318.2 K
Water	1.94 × 10^−7^	3.30 × 10^−7^	5.12 × 10^−7^	7.40 × 10^−7^	1.14 × 10^−6^
EG	6.07 × 10^−4^	7.64 × 10^−4^	9.01 × 10^−4^	1.05 × 10^−3^	1.31 × 10^−3^
PG	8.26 × 10^−4^	9.61 × 10^−4^	1.15 × 10^−3^	1.39 × 10^−3^	1.88 × 10^−3^
MeOH	8.15 × 10^−4^	1.14 × 10^−3^	1.63 × 10^−3^	2.32 × 10^−3^	3.26 × 10^−3^
EtOH	1.73 × 10^−3^	2.65 × 10^−3^	3.98 × 10^−3^	5.81 × 10^−3^	8.37 × 10^−3^
IPA	2.29 × 10^−3^	3.55 × 10^−3^	5.29 × 10^−3^	7.72 × 10^−3^	1.10 × 10^−2^
1-BuOH	2.89 × 10^−3^	4.47 × 10^−3^	6.67 × 10^−3^	9.67 × 10^−3^	1.37 × 10^−2^
2-BuOH	2.94 × 10^−3^	4.54 × 10^−3^	6.74 × 10^−3^	9.78 × 10^−3^	1.38 × 10^−2^
DMSO	2.95 × 10^−3^	3.69 × 10^−3^	4.67 × 10^−3^	5.89 × 10^−3^	7.35 × 10^−3^
EA	4.57 × 10^−3^	5.15 × 10^−3^	6.36 × 10^−3^	7.35 × 10^−3^	8.31 × 10^−3^
THP	6.37 × 10^−3^	8.46 × 10^−3^	1.09 × 10^−2^	1.38 × 10^−2^	1.70 × 10^−2^
PEG-400	9.92 × 10^−3^	1.24 × 10^−2^	1.73 × 10^−2^	2.34 × 10^−2^	3.06 × 10^−2^
*x* ^idl^	3.55 × 10^−2^	3.97 × 10^−2^	4.44 × 10^−2^	4.96 × 10^−2^	5.52 × 10^−2^

^a^ The relative uncertainties *u*_r_ are *u*_r_ (*T*) = 0.011, *u*_r_ (*p*) = 0.003 and *u*_r_ (*x*_e_) = 0.013.

**Table 2:** Different solubility parameters of FBX and several MS at 298.2 K.

Components	Hansen Solubility Parameters				*R*_a_ */MPa^1/2^	∆*δ*/MPa^1/2^	∆*δ* */MPa^1/2^
	*δ*_d_/MPa^1/2^	*δ*_p_/MPa^1/2^	*δ*_h_/MPa^1/2^	*δ*/MPa^1/2^			
FBX	19.30	7.20	6.90	21.70	-	-	-
Water	15.50	16.00	42.30	47.80	37.26	36.67	26.10
EG	18.00	11.10	23.40	31.60	17.15	17.00	9.90
PG	17.40	9.10	21.70	29.20	15.39	15.04	7.50
MeOH	17.40	10.60	22.40	30.30	16.31	15.98	8.60
EtOH	16.20	8.40	17.60	25.40	12.42	11.20	3.70
IPA	15.80	6.60	14.30	22.30	10.24	8.25	0.60
1-BuOH	15.90	6.30	15.20	22.90	10.76	9.01	1.20
2-BuOH	15.80	5.40	12.40	20.80	9.08	6.76	1.10
DMSO	17.40	14.20	7.30	23.60	7.97	7.26	1.90
EA	15.70	5.60	7.00	18.10	7.37	3.94	3.60
THP	16.30	7.20	11.90	21.40	7.81	5.83	0.30
PEG-400	14.60	7.50	9.40	18.90	9.73	5.33	2.80

* These values were calculated between FBX and respective mono solvents.

**Table 3:** Activity coefficients (*γ*_i_) of FBX in several MS at 298.2–318.2 K.

MS	*γ*_i_				
	*T* = 298.2 K	*T* = 303.2 K	*T* = 308.2 K	*T* = 313.2 K	*T* = 318.2 K
Water	183,447	120,446	86,780.2	67,045.2	48,549.3
EG	58.4312	52.0284	49.3229	46.8953	42.1173
PG	42.9883	41.3889	38.5682	35.6282	32.8967
MeOH	43.5463	34.8000	27.1519	21.3567	16.9578
EtOH	20.5244	14.9704	11.1525	8.52961	6.60309
IPA	15.4786	11.1781	8.40559	6.42179	5.00518
1-BuOH	12.2547	8.88315	6.65871	5.12974	4.01502
2-BuOH	12.0608	8.74710	6.59028	5.06955	3.98862
DMSO	12.0201	10.7783	9.52267	8.42330	7.51820
EA	7.76438	7.71898	6.98742	6.74906	6.65091
THP	5.57544	4.70176	4.07784	3.59593	3.23515
PEG-400	3.57786	3.18603	2.55699	2.11563	1.80452

**Table 4:** Apelblat model results for FBX in several MS in terms of model parameters (*A*, *B* and *C*), *R*^2^, and root mean square deviation (*RMSD*).

MS	*A* ± SD	*B* ± SD	*C* ± SD	*R* ^2^	Overall *RMSD* (%)
Water	744.41 ± 5.9712	−41,890 ± 114.24	−108.70 ± 2.5478	0.9983	
EG	−33.013 ± 1.1102	−1829.9 ± 7.8410	5.5734 ± 0.42140	0.9959	
PG	−493.76 ± 3.8412	19,451 ± 44.121	73.966 ± 2.2310	0.9996	
MeOH	−270.82 ± 2.6410	6494.8 ± 52.310	42.461 ± 2.3101	0.9999	
EtOH	323.16 ± 3.2015	−21,470 ± 94.311	−45.198 ± 2.4870	0.9999	
IPA	437.02 ± 3.9104	−26,654 ± 98.410	−62.082 ± 2.5201	0.9999	1.14
1-BuOH	480.91 ± 4.0951	−28,607 ± 102.02	−68.594 ± 2.2810	0.9999	
2-BuOH	502.88 ± 2.0463	−29,579 ± 104.17	−71.876 ± 2.8413	0.9999	
DMSO	−177.93 ± 1.9804	42,067 ± 118.30	27.730 ± 1.0234	0.9998	
EA	57.412 ± 1.2400	−5381.8 ± 101.33	−7.8569 ± 0.53101	0.9919	
THP	460.89 ± 4.0121	−25,357 ± 91.142	−66.855 ± 2.3280	0.9999	
PEG-400	−460.19 ± 3.9904	16,261 ± 94.215	70.385 ± 2.7105	0.9976	

**Table 5:** van’t Hoff model results for FBX in several MS in terms of model parameters (*a* and *b*), *R*^2^, and *RMSD*.

MS	*a* ± SD	*b* ± SD	*R* ^2^	Overall *RMSD* (%)
Water	12.295 ± 1.1024	−8264.2 ± 24.161	0.9974	
EG	4.4757 ± 0.20190	−3539.9 ± 15.411	0.9959	
PG	4.2711 ± 0.18020	−3396.5 ± 14.053	0.9964	
MeOH	15.023 ± 1.1200	−6604.7 ± 21.412	0.9996	
EtOH	18.718 ± 1.1304	−7475.1 ± 23.058	0.9998	
IPA	18.890 ± 1.1322	−7440.7 ± 22.940	0.9996	1.60
1-BuOH	18.926 ± 1.1318	−7381.5 ± 22.664	0.9995	
2-BuOH	18.801 ± 1.1250	−7339.3 ± 22.460	0.9994	
DMSO	8.7471 ± 0.44010	−4347.9 ± 17.184	0.9996	
EA	4.4720 ± 0.19040	−2943.3 ± 12.411	0.9920	
THP	10.648 ± 1.0641	−4778.4 ± 18.722	0.9988	
PEG-400	13.707 ± 1.1200	−5472.1 ± 20.022	0.9965	

**Table 6:** Buchowski-Ksiazaczak *λh* model results for FBX in numerous MS.

MS	*λ* ± SD	*h* ± SD	Overall *RMSD* (%)
Water	3.6910 ± 0.11000	2238.9 ± 10.801	
EG	1.8000 ± 0.0700	1966.6 ± 9.8104	
PG	1.7100 ± 0.06100	1986.2 ± 9.9420	
MeOH	0.44800 ± 0.03100	14742 ± 8.1200	
EtOH	2.3539 ± 0.09200	3175.5 ± 13.710	
IPA	2.5966 ± 0.10100	2865.5 ± 12.840	2.86
1-BuOH	2.7544 ± 0.10300	2679.8 ± 11.510	
2-BuOH	2.7161 ± 0.10210	2702.1 ± 11.940	
DMSO	−0.81040 ± 0.04100	−5365.1 ± 19.120	
EA	0.57760 ± 0.03000	5095.7 ± 18.052	
THP	0.03210 ± 0.00000	145,744 ± 128.514	
PEG-400	1.4601 ± 0.05101	3747.7 ± 14.501	

**Table 7:** Apparent thermodynamic parameters (Δ_sol_*H*^0^, Δ_sol_*G*^0^, and Δ_sol_*S*^0^) along with *R*^2^ values for FBX in numerous MS ^a^.

MS	Δ_sol_*H*^0^/kJ mol^−1^	Δ_sol_*G*^0^/kJ mol^−1^	Δ_sol_*S*^0^/J mol^−1^ K^−1^	*R* ^2^
Water	68.79	37.21	102.5	0.9973
EG	29.46	17.96	37.34	0.9958
PG	28.27	17.29	35.64	0.9966
MeOH	54.98	16.43	125.1	0.9996
EtOH	62.23	14.20	155.9	0.9997
IPA	61.94	13.47	157.3	0.9995
1-BuOH	61.45	12.89	157.6	0.9994
2-BuOH	61.10	12.86	156.5	0.9994
DMSO	36.19	13.74	72.89	0.9996
EA	24.50	13.01	37.30	0.9921
THP	38.94	11.62	88.70	0.9987
PEG-400	45.56	10.38	114.1	0.9966

^a^ The relative uncertainties are *u* (Δ_sol_*H*^0^) = 0.033, *u* (Δ_sol_*G*^0^) = 0.044 and *u* (Δ_sol_*S*^0^) = 0.047.

## Data Availability

This study did not report any data.

## Supplementary Materials

The following supporting information can be downloaded at: https://www.mdpi.com/article/10.3390/molecules27134043/s1. Figure S1: Correlation of experimental solubilities of FBX with “van’t Hoff model” in different mono solvents as a function of 1/*T*; symbols represent the experimental solubility values of FBX and the solid lines represent the solubility data calculated by “van’t Hoff model”; Table S1: List of materials.

## Sample Availability

Samples of the compounds FBX are available from the authors.
