# Supplementary material for: Solubility and Thermodynamic Data of Febuxostat in Various Mono Solvents at Different Temperatures
Source: Molecules. 2022 Jun 23;27(13):4043. doi: 10.3390/molecules27134043 (PMC9268579; doi:10.3390/molecules27134043)
Supplement: Supplementary file 1 [file molecules-27-04043-s001.zip › molecules-1784670-supplementary.pdf]

Supplementary Materials

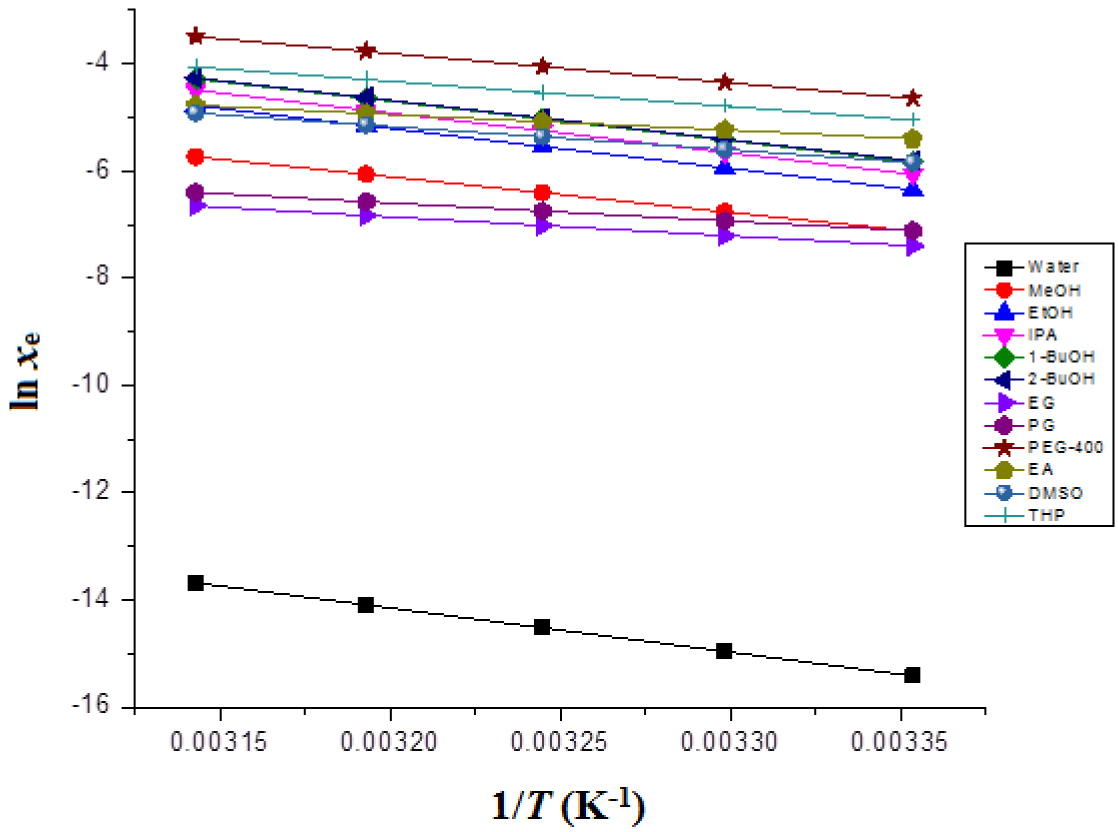

Figure S1. Correlation of experimental solubilities of FBX with "van't Hoff model" in different mono solvents as a function of 1/*T*; symbols represent the experimental solubility values of FBX and the solid lines represent the solubility data calculated by "van't Hoff model".

Table S1. List of materials.

Material	Molecular formula	Molar mass (g mol^{-1})	CAS Registry no.	Purification method	Mass fraction purity	Analysis method	Source
FBX	$C_{16}H_{16}N_2O_3S$	316.40	144060-53-7	None	>0.99	HPLC	E-Merck
MeOH	CH_3OH	32.04	67-56-1	None	>0.99	GC	E-Merck
EtOH	C_2H_5OH	46.07	64-17-5	None	>0.99	GC	E-Merck
IPA	C_3H_8O	60.10	67-63-0	None	>0.99	GC	E-Merck
1-BuOH	$C_4H_{10}O$	74.12	71-36-3	None	>0.99	GC	E-Merck
2-BuOH	$C_4H_{10}O$	74.12	78-92-2	None	>0.99	GC	E-Merck
EG	$C_2H_6O_2$	62.07	107-21-1	None	>0.99	GC	Sigma Aldrich
PG	$C_3H_8O_2$	76.09	57-55-6	None	>0.99	GC	Sigma Aldrich
PEG-400	$H(OCH_2CH_2)_nOH$	400	25322-68-3	None	>0.99	HPLC	Sigma Aldrich
THP	$C_6H_{14}O_3$	134.17	111-90-0	None	>0.99	GC	Gattefosse
DMSO	C_2H_6OS	78.13	67-68-5	None	>0.99	GC	Sigma Aldrich
EA	$C_4H_8O_2$	88.11	141-78-6	None	>0.99	GC	Sigma Aldrich
Water	H_2O	18.07	7732-18-5	None	-	-	Milli-Q

Both the analysis method and purity were provided by supplier of each material
